# Cognitive Impairment of Patient With Neurological Cerebrovascular Disease Using the Artificial Intelligence Technology Guided by MRI

**DOI:** 10.3389/fpubh.2021.813641

**Published:** 2022-03-03

**Authors:** Lifang Zhang, Yanran Li, Lin Bian, Qingrong Luo, Xiaoxi Zhang, Bing Zhao

**Affiliations:** ^1^Department of Neurology, Changzhi People's Hospital, Changzhi Medical College, Changzhi, China; ^2^Department of Mental Health, Changzhi Medical College, Changzhi, China; ^3^Department of Radiology, First Affiliated Hospital of Xinjiang Medical University, Ürümqi, China

**Keywords:** artificial intelligence technology, cognitive impairment, MRI, neuropsychological assessment, neurological cerebrovascular disease

## Abstract

This study was to explore the application of MRI based on artificial intelligence technology combined with neuropsychological assessment to the cognitive impairment of patients with neurological cerebrovascular diseases. A total of 176 patients were divided into a control group, a vascular cognitive impairment non-dementia (VCIND) group, a vascular dementia (VD) group, and an Alzheimer's disease (AD) group. All patients underwent MRI and neuropsychological evaluation and examination, and an improved fuzzy C-means (FCM) clustering algorithm was proposed for MRI processing. It was found that the segmentation accuracy (SA) and similarity (KI) data of the improved FCM algorithm used in this study were higher than those of the standard FCM algorithm, bias-corrected FCM (BCFCM) algorithm, and rough FCM (RFCM) algorithm (*p* < 0.05). In the activities of daily living (ADL), the values in the VCIND group (23.55 ± 6.12) and the VD group (28.56 ± 3.1) were higher than that in the control group (19.17 ± 3.67), so the hippocampal volume was negatively correlated with the ADL (*r* = −0.872, *p* < 0.01). In the VCIND group (52.4%), VD group (31%), and AD group (26.1%), the proportion of patients with the lacunar infarction distributed on both sides of the brain and the number of multiple cerebral infarction lesions (76.2, 71.4, and 71.7%, respectively) were significantly higher than those in the control group (23.9 and 50%). In short, the improved FCM algorithm showed a higher segmentation effect and SA for MRI of neurological cerebrovascular disease. In addition, the distribution, number, white matter lesions, and hippocampal volume of lacunar cerebral infarction were related to the cognitive impairment of patients with cerebrovascular diseases.

## Introduction

Cerebrovascular disease is one of the main factors to cause vascular cognitive impairment (VCI) (1, 2). With the aging of current society, the incidence of cerebrovascular disease and VCI has increased significantly, ranking second only to Alzheimer's disease (AD), and is an important cause of the cognitive decline in the elderly (3). VCI is a common clinical type. It is the same as other cognitive diseases, and its clinical characteristics are gait, mood, behavior, and urination disorders (4). When the early cognitive impairment occurs, the condition will be mild so that it will often be ignored by others. When it develops into a disorder or even dementia, it is already too late and is not easy to diagnose and treat (5, 6). Therefore, it is very important to detect and find the vascular dysfunction as early as possible.

At present, the diagnosis of VCI is mainly based on the neuropsychological assessment to measure the cognitive function of the patients and to determine the relationship between cognitive impairment and brain disease. If the computer MRI is used as an auxiliary diagnostic method, the diagnosis results will be more reliable (7). With the continuous increase in the breadth and depth of clinical applications of medical images, medical images have become one of the important research directions in medical image processing. The technical difficulty of the brain MRI segmentation is mainly reflected in the following aspects. (1) Due to the principle and technical limitations of MRI and the influence of various factors in the image acquisition process, the image has partial volume effects, field offset effects, noise, motion artifacts, etc. (8, 9). (2) The tissue structure of the brain is complex. Normal tissues in the brain MRI mainly include the cerebral cortex, gray matter, white matter, cerebrospinal fluid, etc.; and the diseased tissues mainly include tumors, edema, and necrosis and cystic transformation within the tumors, and each type of tissue structure is newly transmitted and complex. (3) The space occupation, new transmission, and size of intracranial lesions are uncertain; the gray distribution between different soft tissues or between the diseased tissue and the soft tissue is uneven; and the density distribution is aliased, which is very convenient and very fuzzy. (4) There are big differences in the organizational results of different individuals, even the same person at different ages will have great differences (10–12). Therefore, the automatic segmentation technology using brain tissue MRI plays an important role in the diagnosis and treatment of brain diseases. MRI is the most widely used technique in radio imaging. As a dynamic and flexible technique, MRI can use different parameters for imaging, including longitudinal relaxation time (T1), lateral relaxation time (T2), T1 weighting imaging (T1WI), T2 weighting imaging (T2WI), etc. (13, 14). The imaging of these different parameters can effectively use brain MRI segmentation. Therefore, this study adopts an improved fuzzy C-means (FCM) clustering algorithm to overcome the shortcomings of FCM itself. The clustering-based segmentation method is used to divide the image pixels into several categories according to their similarity. The distance between different individuals and the distance between classes are larger, and the distance between individuals of the same class is smaller. FCM assigns each pixel to each type of organization with a different degree of membership and then has a certain criterion to uniquely divide each pixel into a certain type of organization to obtain the result of segmentation (15, 16).

Therefore, the relevant case data and MRI data of the patients in the Department of Neurology and Physical Examination, Changzhi People's Hospital are selected. In this study, lacunar cerebral infarction, white matter lesions, and hippocampal volume of patients with neurological cerebrovascular disease were studied. Through MRI based on artificial intelligence technology, the neuropsychological evaluation was combined to analyze its correlation with cognitive impairment, aiming to provide a reliable theoretical basis for early diagnosis and the detection, diagnosis, and treatment of vascular cognitive impairment.

## Materials and Methodology

### Information of Research Objects

The relevant case data and MRI data of the patients in the Department of Neurology and Physical Examination, Changzhi People's Hospital from October 1, 2018 to February 1, 2020 were selected. The studies involving human participants were reviewed and approved by Changzhi People's Hospital Ethics Committee. The patients/participants provided their written informed consent to participate in this study. The patients were divided into a control group, a vascular cognitive impairment non-dementia (VCIND) group, a vascular dementia (VD) group, and an AD group according to the Clinical Dementia Rating (CDR) Scale. The classification and diagnostic criteria of VD in the vascular cognitive impairment in China in 2019 were met in the VD group, and the core clinical criteria in the diagnosis of AD revised by the NIA-AA in 2011 were met in the AD group (13). There were 42, 42, and 46 cases in the VCIND, VD, and AD groups, respectively. About 46 cases of healthy patients without memory loss in the Department of Physical Examination were included in the control group, and they had no other related diseases that may affect their cognitive function.

The inclusion criteria: first, completed clinical case and MRI data; second, no acute cerebrovascular disease in at least 1 year; third, meeting the criteria of VD and AD in the diagnostic guidelines for the diagnosis and treatment of vascular cognitive impairment in China Diagnostic criteria for vascular VCI; and forth, no cerebral white matter lesions caused by hydrocephalus, cerebral hemorrhage shown in the MRI.

Exclusion criteria: first, more than 50% of the cranial vascular narrowing shown in the intracranial vascular ultrasonography, head MRI, or head CT angiography; second, having abnormal neurological diseases such as Parkinson's, brain tumors, and encephalitis; third, moderate aphasia, depression, or mental disorders, and other case being unable to complete the investigation; and forth, hereditary small blood vessel disease.

### Neuropsychological Assessment

The patients were performed with relevant questions and answers for the test and evaluation in a quiet environment. The test tables included the mini-mental state examination (MMSE), montreal cognitive assessment (MOCA), memory and executive screening (MES), activities of daily living (ADL), and CDR (14). The neuropsychological assessment was evaluated by an experienced neurologist in the neuropsychological assessment.

### MRI Examination Based on the Artificial Intelligence Technology

The MRI examination: the Siemens verio3.0T magnetic resonance scanner was used to scan the patient's head for conventional transverse, axial T2WI, sagittal T1WI, coronary T2 fluid attenuation inversion recovery sequence (FLAIR), and hippocampal volume scan. The results of the MRI examination were identified by 2–3 imaging experienced physicians. For patients being unable to cooperate with the MRI examination temporarily, oral chloral hydrate could be used for sedation.

### Improved FCM Image Segmentation Algorithm

The correct segmentation of brain tissue is an important prerequisite for the location and description of lesions. Noise and offset fields, as important factors affecting the quality of brain MRI, often exist at the same time. Therefore, image segmentation algorithms that can remove noise and offset fields are very important for the correct division of brain tissue (17). Therefore, based on the FCM algorithm, an MRI segmentation algorithm that removed noise and corrected the offset field was proposed.

The actual image would be affected by additive noise and multiplicative offset field, which can be expressed as follows:


(1) $$\begin{array}{l} q_{i}=a_{i}p_{i}+c \end{array}$$

In Equation (1), *q*_i_ represents a noisy image, *a*_*i*_ represents an offset field, *p*_*i*_ represents an uncontaminated image, and *c* represents noise. In actual operation, both the offset field and the uncontaminated image are unknown parameters, so the offset field was regarded as a slowly changing and smooth uneven field.

The traditional coherent local intensity clustering (CLIC) algorithm based on the gray correction can be used to evaluate the local neighborhood *M*_i_ of the offset field, then below equation could be obtained:


(2) $$\begin{array}{l} M_{i}=\left\{j:|j-i|\leq r\right\} \end{array}$$

In Equation (2), *r* represents the radius of the neighborhood. Taking into account the slow change of the offset field, *a*_*j*_ can be replaced by *a*_*i*_ in a small range, so the *q*_*j*_ belonging to the l-th category can be expressed as follows:


(3) $$\begin{array}{l} q_{j}=a_{j}d_{l}+c \end{array}$$

The cost function of pixel *q*_*i*_ can be expressed as follows:


(4) $$\begin{array}{l} \lambda_{i}=\overset{m}{\underset{l=1}{\sum }}\underset{j\epsilon M_{i}}{\sum }{{\mu_{l}}_{j}^{m}K\left(i-j\right)||q_{i}-a_{i}d_{l}||}^{2} \end{array}$$

In Equation (4), λ_*i*_ represents the cost function, and *K*(*i* – *j*) represents the weight coefficient, and Equation (5) could be obtained:


(5) $$K\left(i-j\right)=\{\begin{array}{cc} \frac{e^{-{\left|i-j\right|}^{2}}}{\nu }/2\sigma^{2} & \left|i-j\right|\leq r\\ 0 & other \end{array}\;$$

The total cost function of the CLIC algorithm can be combined with the FCM algorithm to get the equation below.


(6) $$\begin{array}{l} T_{c}=\overset{m}{\underset{i=1}{\sum }}\overset{d}{\underset{l=1}{\sum }}\underset{j\epsilon M_{i}}{\sum }{{\mu_{l}}_{j}^{m}K\left(i-j\right)||q_{i}-a_{i}d_{l}||}^{2}\; \end{array}$$

In Equation (6), *T*_*C*_ is the total cost function. The CLIC algorithm can directly process the original data, and the obtained offset field is smooth and changes slowly, without subsequent filtering operations. However, because the objective function does not consider the constraints of domain information, it is more sensitive to noise parameters. In this paper, the spatial information is added to the objective function of the CLIC algorithm, and a new weight coefficient *R*_*li*_ is proposed, which is as follows:


(7) $$\begin{array}{l} T_{CR}=\overset{m}{\underset{i=1}{\sum }}\overset{d}{\underset{l=1}{\sum }}\underset{j\epsilon M_{i}}{\sum }{\mu_{l}}_{j}^{m}\left[K\left(i-j\right)||q_{i}-a_{i}d_{l}|{|}^{2}+R_{li}\right] \end{array}$$

In Equation (7), *m* indicates the existence of *m* pixels in the image, *d* indicates the classification of the image into *d* categories, and μ_*lj*_ represents the degree to which pixel *j* belongs to *d* category. Then the calculated equations for cluster center, membership degree, and offset field can be given as follows:


(8) $$\begin{array}{l} \mu_{lj}=\frac{1}{{\sum_{f=1}^{d}\left[\frac{W_{lj}+R_{li}}{W_{fj}+R_{fj}}\right]}^{\frac{1}{n-1}}} \end{array}$$

(9) $$\begin{array}{l} W_{lj}=\overset{m}{\underset{i=1}{\sum }}K\left(i-j\right)||q_{i}-a_{i}d_{l}\;|{|}^{2} \end{array}$$

(10) $$\begin{array}{l} CC_{l}=\frac{\sum_{j=1}^{m}{\mu_{l}}_{j}^{m}q_{j}\sum_{i=1}^{m}K\left(i-j\right)a_{i}}{\sum_{j=1}^{m}{\mu_{l}}_{j}^{m}q_{j}\sum_{i=1}^{m}K\left(i-j\right)a_{i}^{2}} \end{array}$$

(11) $$\begin{array}{l} a_{i}=\frac{\sum_{i=1}^{m}K\left(i-j\right)q_{j}\sum_{j=1}^{m}{\mu_{l}}_{j}^{m}d_{l}}{\sum_{i=1}^{m}K\left(i-j\right)q_{j}\sum_{j=1}^{m}{\mu_{l}}_{j}^{m}d_{l}^{2}} \end{array}$$

**Figure 1:**
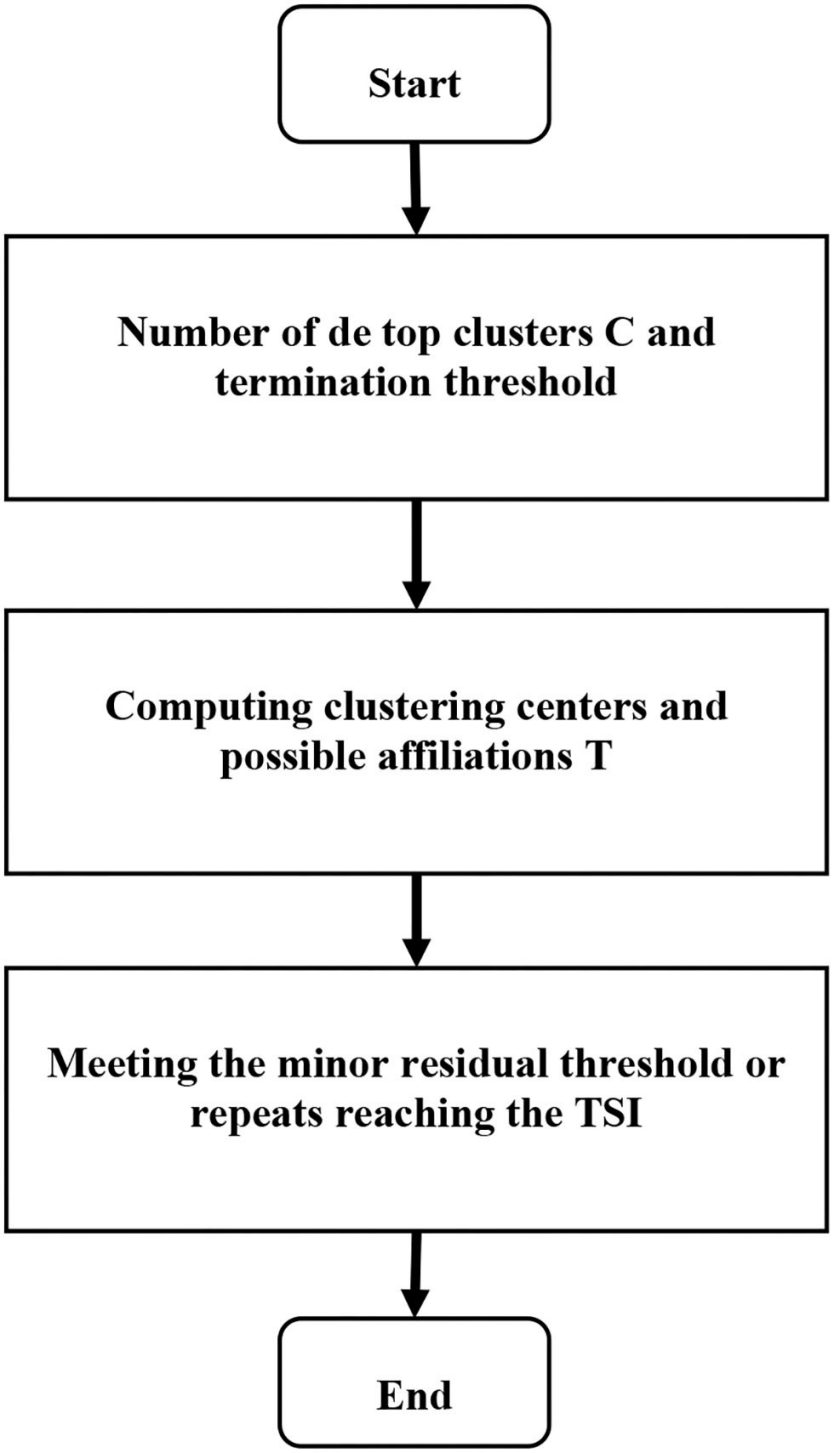
Process of MRI segmentation using the fuzzy C-means clustering (FCM) algorithm.

Equations (9–11) represent the cluster center, degree of membership, and offset field, respectively. *m* represents the existence of *m* pixels in the image, *d* represents the classification of the image into *d* categories, and μ_*lj*_ represents the degree to which pixel *j* belongs to *d* category. Based on the possibility of the FCM algorithm MRI segmentation method, the steps were as follows. Step 1: the noise and brain tissue in the brain MRI were removed, and the image was normalized to prepare for the next step. Step 2: the histogram statistics was performed on the three-dimensional MRI data, and the value of 0.707 near the highest value sample was automatically obtained, and the threshold was set to obtain the classification number. Step 3: the initial segmentation was performed through FCM with an uncertain membership relationship to obtain the initial membership degree and cluster center. Step 4:the matrix size of multiple own membership degrees was adjusted. Step 5: the collaborative possibility FCM algorithm was adopted to segment the lesion area to obtain the segmented image. Therefore, the calculation process of the algorithm constructed in this study can be shown in Figure 1.

### Assessment of Lacunar Infarction

An MRI examination of the patients' brains was performed. When the sagittal T1WI phase was in a low-signal state, and the axial T2WI and coronary T2 FLAIR were in a high-signal state, it was determined that the diameter was less than 25 mm, and the ischemic infarct lesion is determined. When the diameter was less than 3 mm, it was considered to increase the peripheral blood vessel gap. The numbers of cerebral infarction lesions with a diameter of 3–25 mm were recorded, and the average value was taken as the number of cerebral infarction lesions. The identification and counting were carried out by an experienced radiologist.

### Evaluation of White Matter Lesions

An MRI examination of the patients' brains was performed. The T1 weighted was in a low-signal state in the white matter in the bilaterally symmetrical lateral ventricle, whereas the T2 weighted and FLAIR sequence were in a high-signal state with patchy shadows and blurred boundaries. It was determined as the white matter lesion (15). Classification of the white matter lesion was as below: (Level 0: no lesion; Level 1: punctate lesion; Level 2: fusion of lesion; Level 3 lesion merging in large areas).

In this study, segmentation accuracy (SA) and similarity (KI) were used as indicators to evaluate segmentation results. The available equations were given as follows:


(12) $$\begin{array}{l} KI=\frac{2\left(A⋂B\right)}{2\left(A⋂B\right)+\left(A-B\right)+\left(B-A\right)} \end{array}$$

(13) $$\begin{array}{l} SA=\overset{c}{\underset{i=1}{\sum }}\frac{A_{i}⋂B_{i}}{\sum_{j=1}^{c}B_{j}} \end{array}$$

In Equations (12) and (13), A represents the target area pixels obtained by the algorithmic segmentation, and B represents the target area pixels obtained by the standard segmentation.

### Statistical Method

In this study, all the experimental data were analyzed by using the SPSS 13.0. The percentage (%) and the mean and SD ($\bar{x}$ ± s) were used to indicate the measured data. The χ^2^ test was used to compare the gender, age, and education level of patients in the control, VCIND, VD, and AD groups, and the *t*-test was used to compare the age among four groups. The SPSS Spearman correlation analysis was used to analyze the correlation between the hippocampal volume and age, education level, and ADL, and to understand the relationship of the data. Comparison of data in different groups was analyzed by the one-way ANOVA. If *p* < 0.05, the difference had a statistical significance.

## Results

### MRI of the Patients

**Figure 2:**
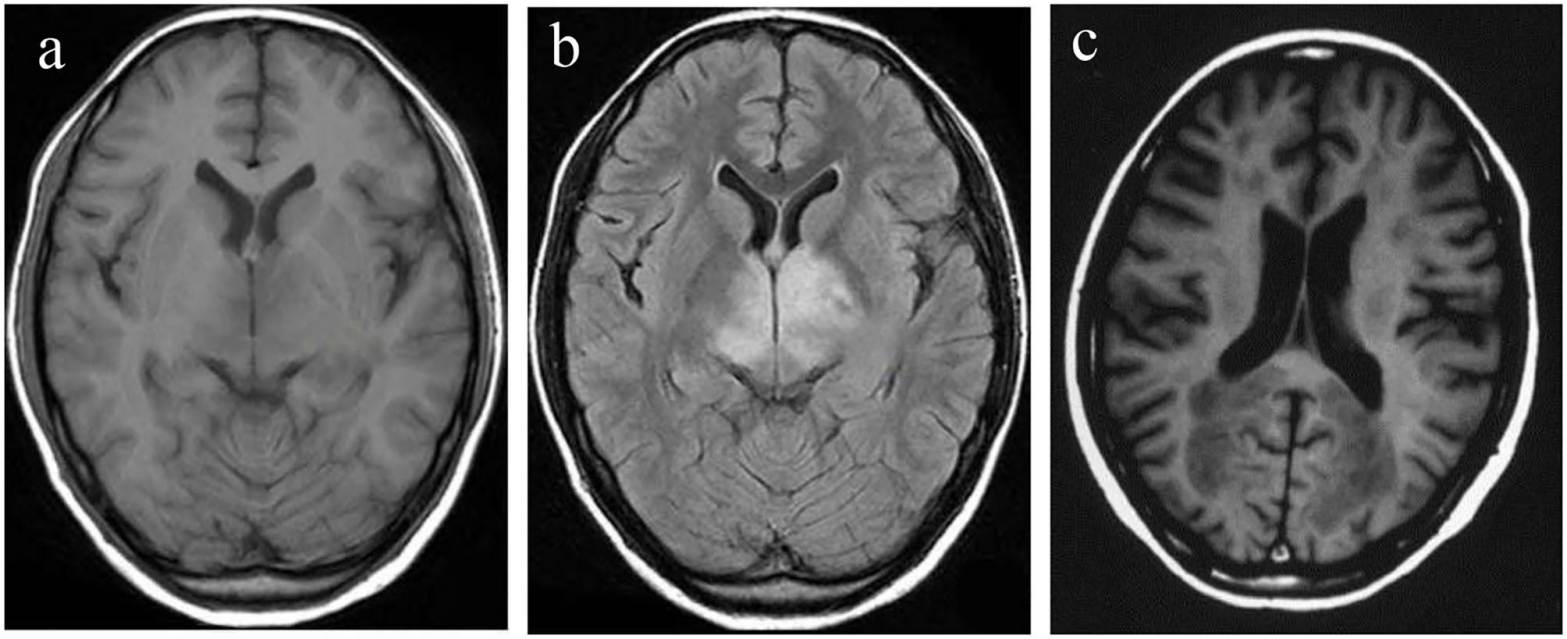
Head MRI of the patients in control, VD, and AD groups. **(a)** Control group; **(b)** VD group; **(c)** AD group. AD, Alzheimer's disease; VD, vascular dementia.

The head MRI of the patients in the control, VD, and AD groups were compared. There was no lacunar infarction, white matter lesions, and hippocampal volume changes for the patients in the control group, as shown in Figure 2a. The white matter lesion occurred for the patients in the VD group, as shown in Figure 2b. The patients in the AD group had lacunar infarction, white matter lesions, and reduced hippocampal volume, as shown in Figure 2c.

### Comparison of Algorithm Segmentation Effect

**Figure 3:**
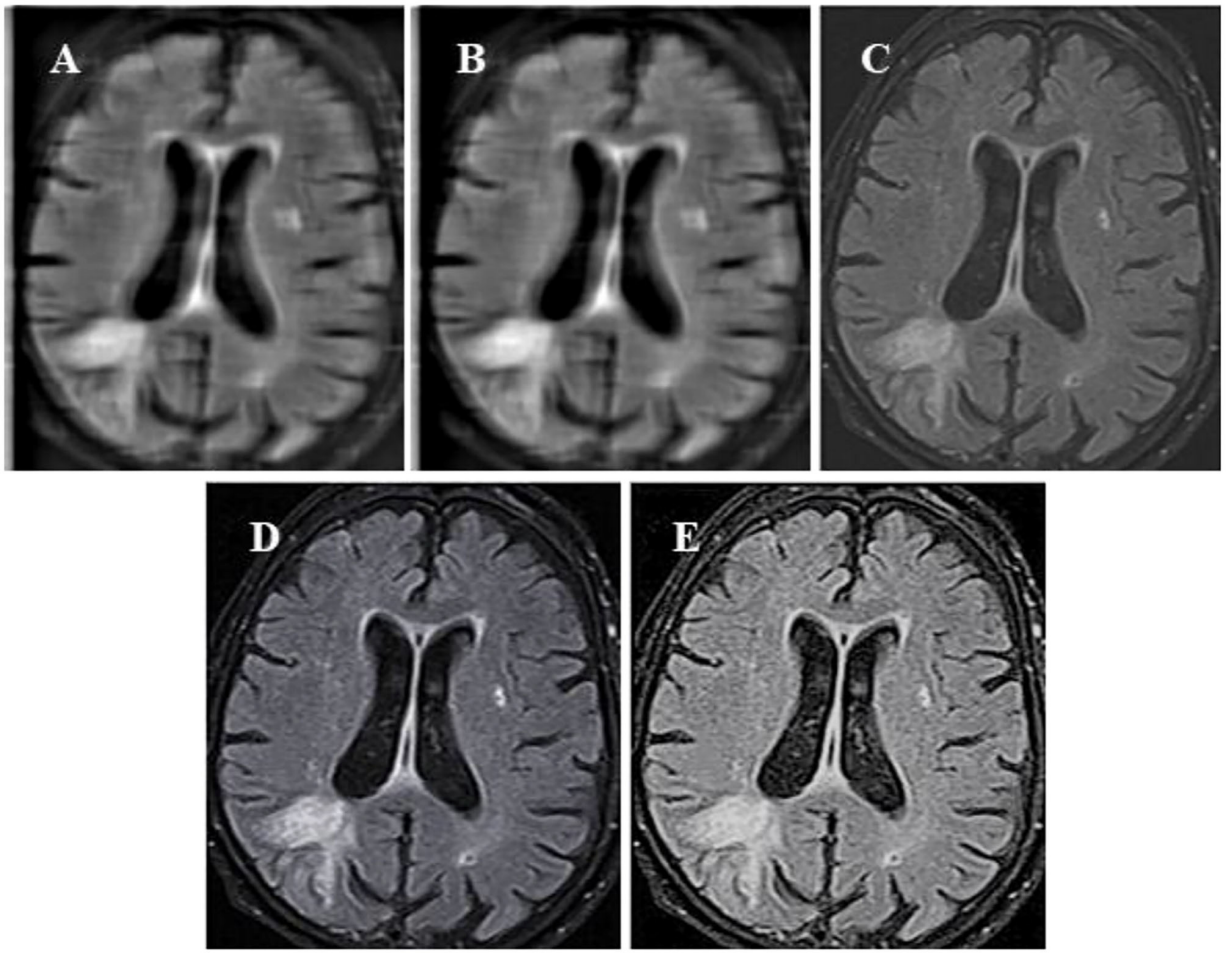
Segmentation results of MRI of brain tumors by the algorithm. **(A)** The original image; **(B)** the standard FCM algorithm; **(C)** the RFCM algorithm; **(D)** the BCFCM algorithm; and **(E)** the improved FCM algorithm. FCM, fuzzy C-means; BCFCM, bias-corrected fuzzy C-means; RFCM, rough fuzzy C-means.

In this study, the standard FCM algorithm was introduced, the bias-corrected FCM (BCFCM) improved based on the global information, and compared the segmentation effect based on the rough FCM (RFCM) algorithm were compared with the algorithm proposed in this study. As shown in Figure 3, the improved FCM algorithm used in this study was significantly better than other algorithms in terms of noise removal and detail preservation, and the segmentation effect was the most ideal.

**Figure 4:**
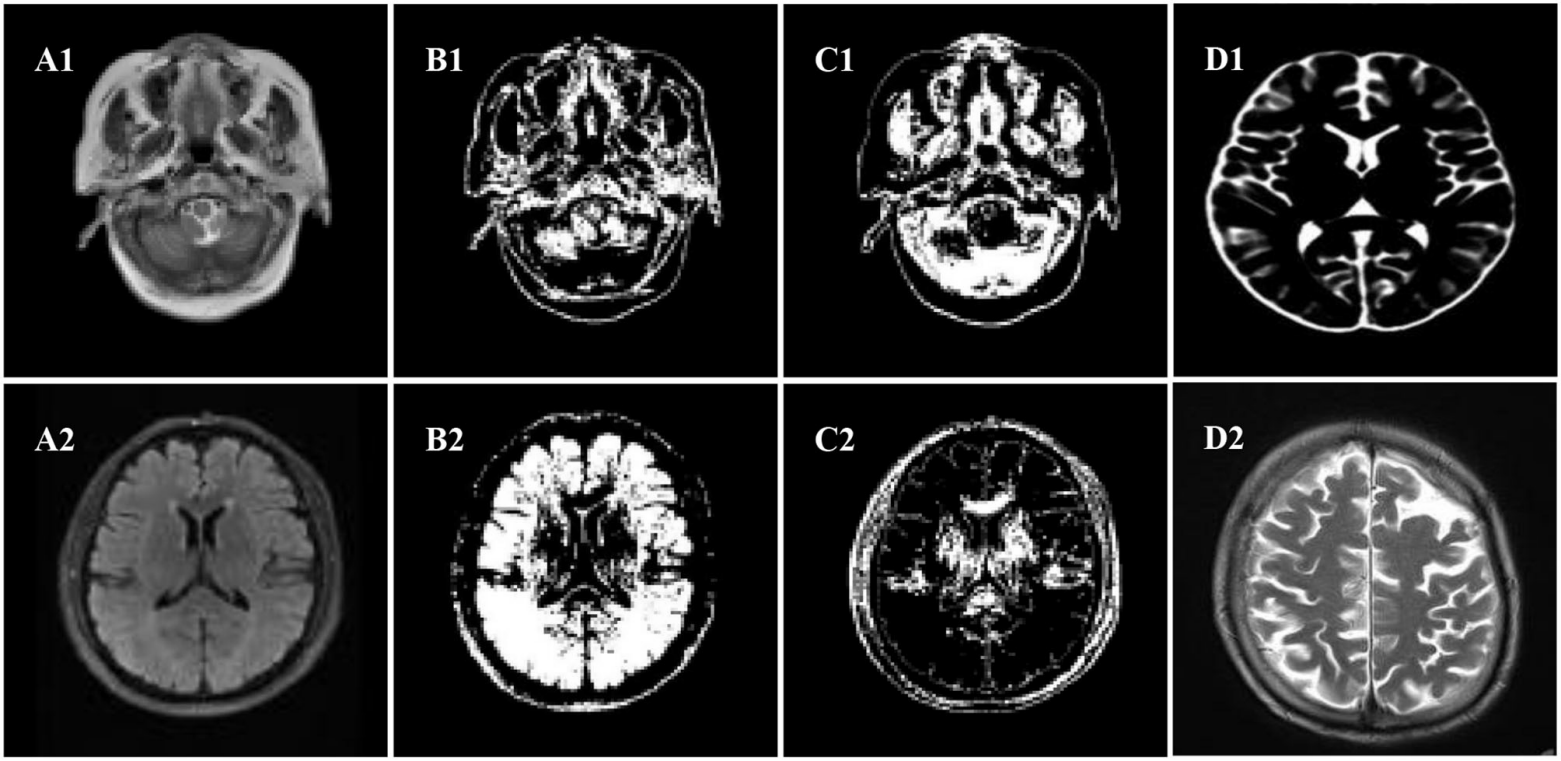
Segmentation effect diagram. **(A1,A2)** Original images, **(B1,B2)** white matter, **(C1,C2)** gray matter, **(D1,D2)** organ.

Normal tissues in the brain MRI mainly included cerebral cortex, gray matter, white matter, cerebrospinal fluid, etc.; the diseased tissues mainly included tumors, edema, and necrosis and cystic transformation inside tumors, and the structure of each tissue was newly transmitted and complex. The space occupancy, new transmission, and size of intracranial lesions were uncertain; the gray distribution between different soft tissues or between the diseased tissue and the soft tissue was uneven; and the aliased density distribution and other factors would affect the image segmentation effect. The MRI of the patient's brain was processed by an algorithm, and the comparison of the white matter, gray matter, and organs of the brain was shown in Figure 4.

**Figure 5:**
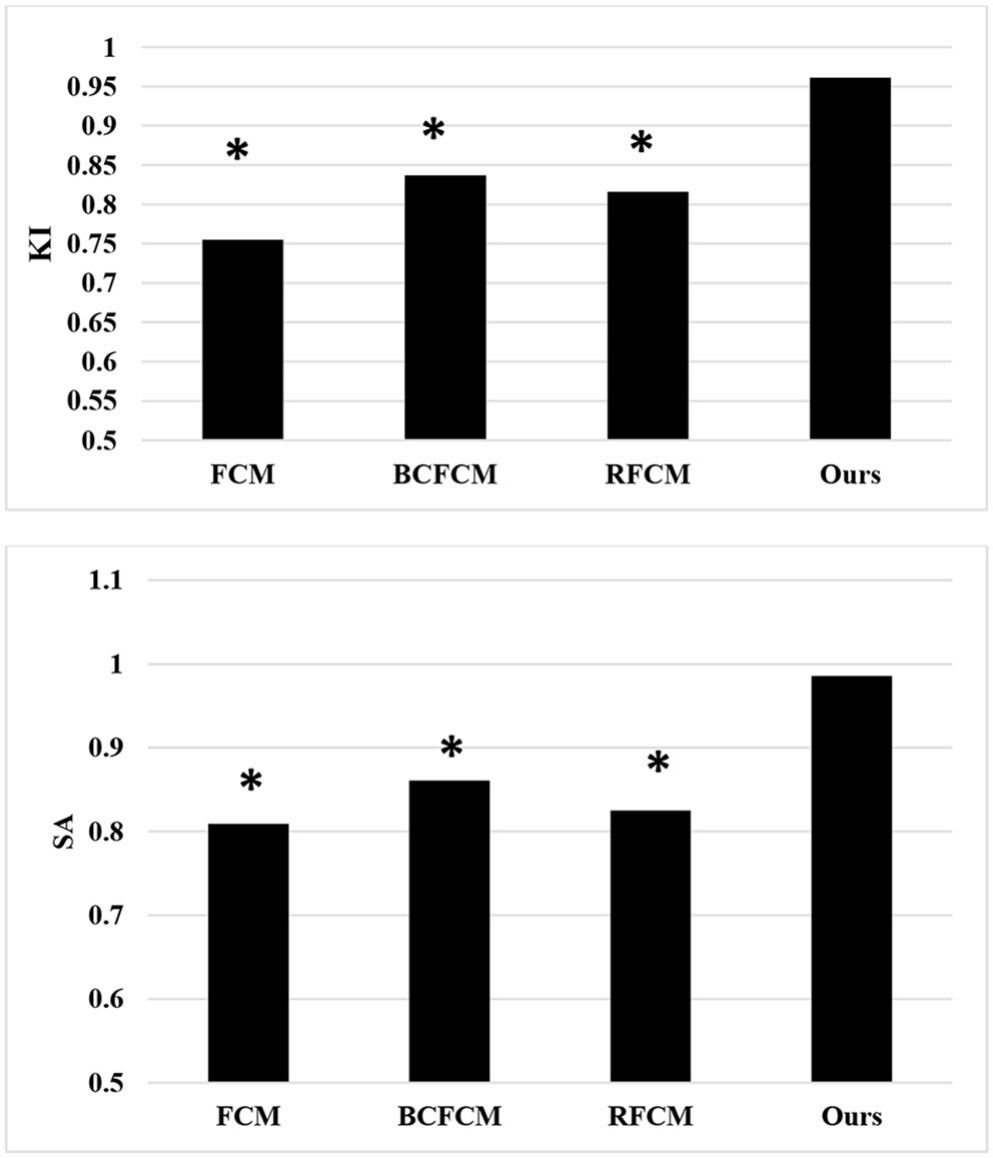
Comparison of segmentation indexes of algorithms. *The difference compared with the algorithm in this study was statistically significant (*p* < 0.05).

Further comparison of the quantitative data results (Figure 5) showed that the KI and SA of the improved FCM algorithm used in this study were 0.961 and 0.986, respectively; those of the FCM algorithm were 0.755 and 0.809, respectively; those of the BCFCM algorithm were 0.837 and 0.861, respectively; and those of the RFCM algorithm were 0.816 and 0.825, respectively. The KI and SA index data of the improved FCM algorithm used in this study were significantly higher than the standard FCM algorithm, BCFCM algorithm, and RFCM algorithm, showing statistically significant differences (*p* < 0.05).

### Comparison of Patients' Basic Information

**Figure 6:**
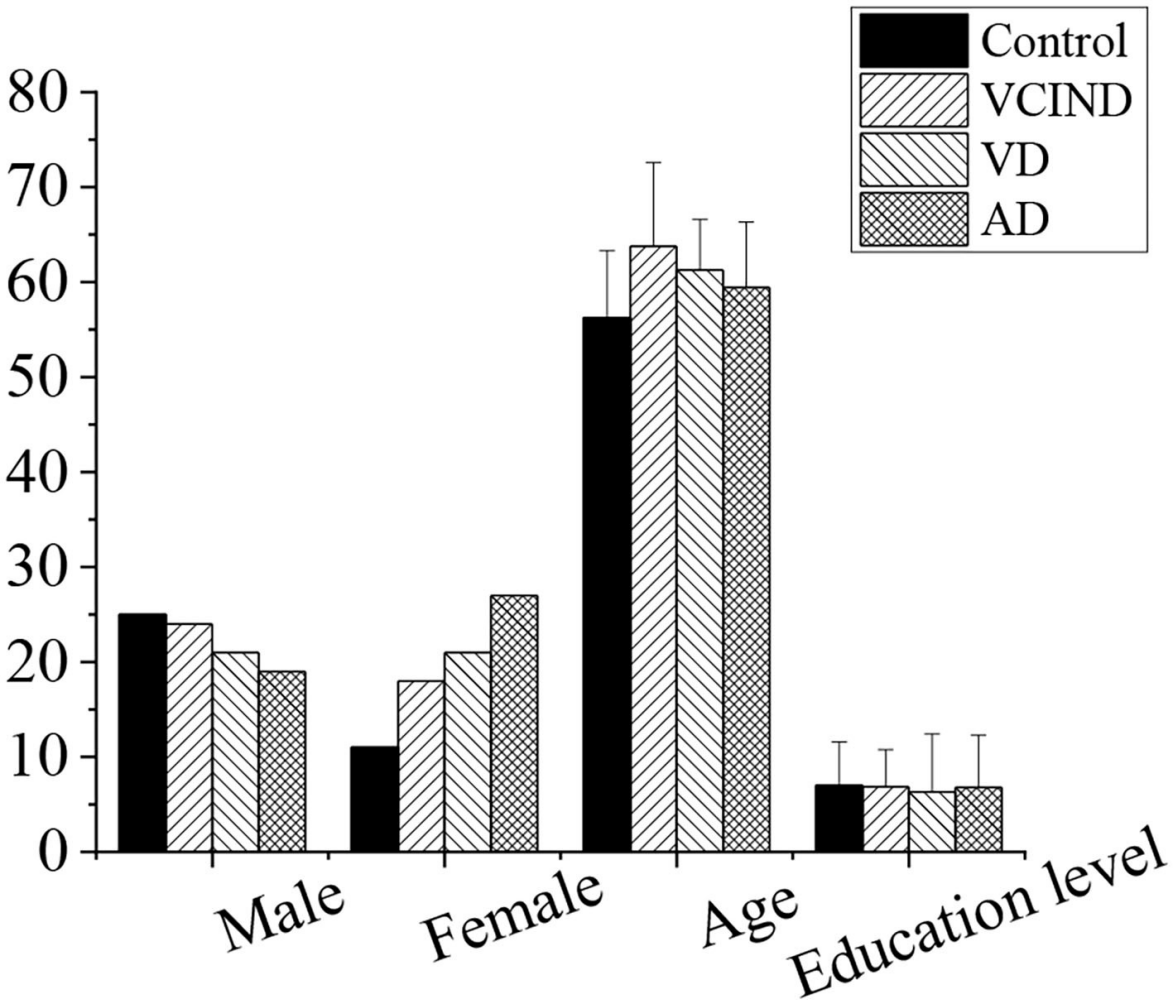
Comparison of patients' basic information in the control, VCIND, VD, and AD groups. There was no statistic difference for the gender, age, and education level (*p* > 0.05). AD, Alzheimer's disease; VCIND, vascular cognitive impairment non-dementia; VD, vascular dementia.

The sex and education level of the patients in the control, VCIND, VD, and AD groups were compared, as shown in Figure 6. It was found that there was no significant difference in gender and education level by the χ^2^ test (*p* > 0.05), and there was no significant difference in age by the *t*-test (*p* > 0.05). After analysis of the patient's basic information and data, it was comparable in gender, age, and educational level (*p* > 0.05).

### Analysis of Related Risk Factors of Patients in Four Groups

**Figure 7:**
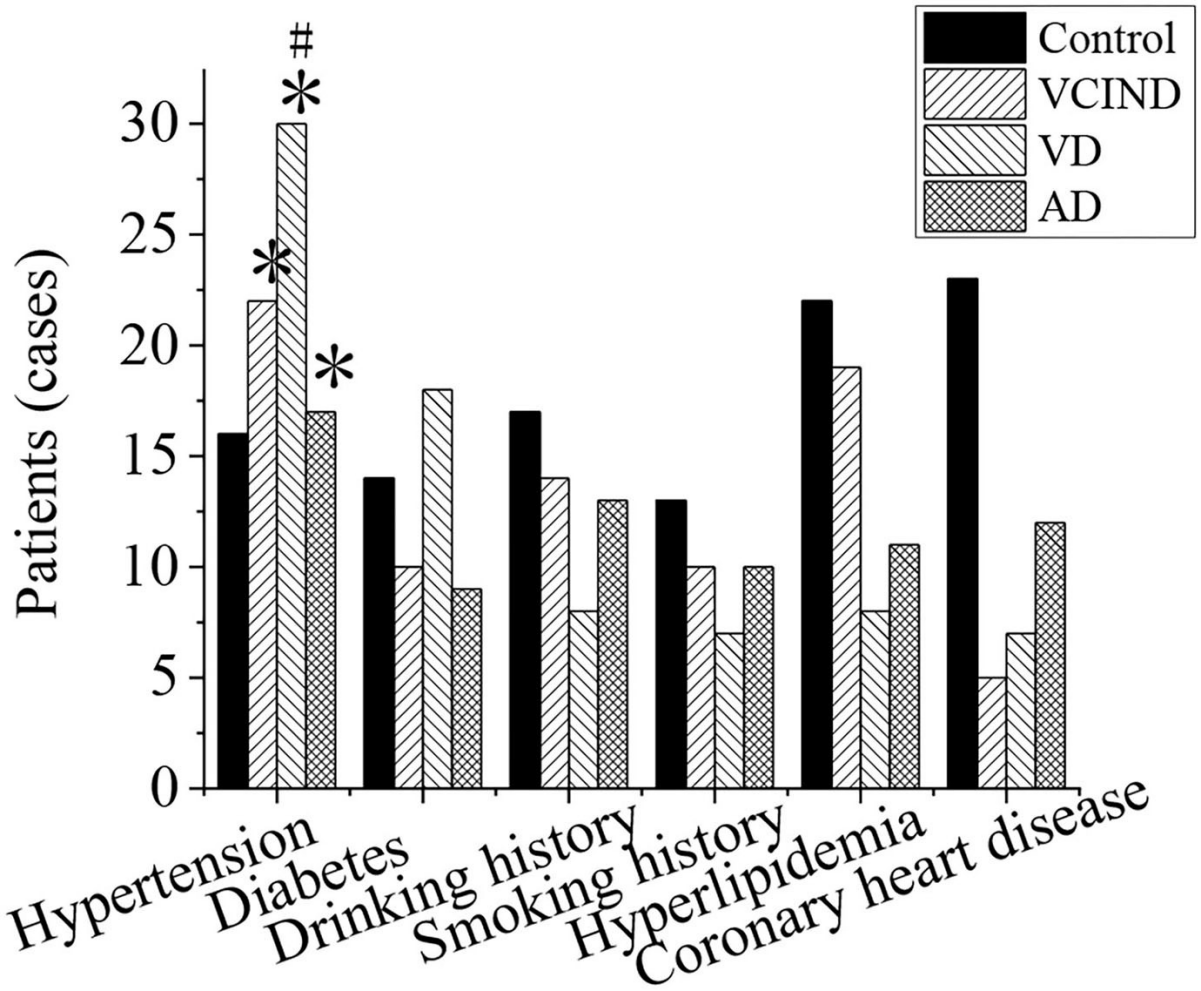
Comparison of related risk factors of patients in the control, VCIND, VD, and AD groups. *p < 0.05 if compared with the control group, whereas ^#^*p* < 0.05 if compared with the VCIND group. AD, Alzheimer's disease; VCIND, vascular cognitive impairment non-dementia; VD, vascular dementia.

As shown in Figure 7, the hypertension, diabetes, smoking history, drinking history, hyperlipidemia, and coronary heart disease of the patients in the control group (46 cases), VCIND group (42 cases), VD group (42 cases), and AD group (46 cases) were analyzed and compared. It was found that the number of patients with associated hypertension in the VCIND group (22 cases, 52.4%), VD group (30 cases, 71.4%), and AD group (17 cases, 37%) was significantly higher than that in the control group (16 cases, 34.8%) with a statistical difference (^*^p < 0.05). The VD group was significantly higher than that in the VCIND group, which had a statistical difference by comparison (^#^*p* < 0.05). There were no significant statistical differences among the other related risk factors (*p* > 0.05).

### Evaluation of Cognitive Function of the Patients in Four Groups

**Figure 8:**
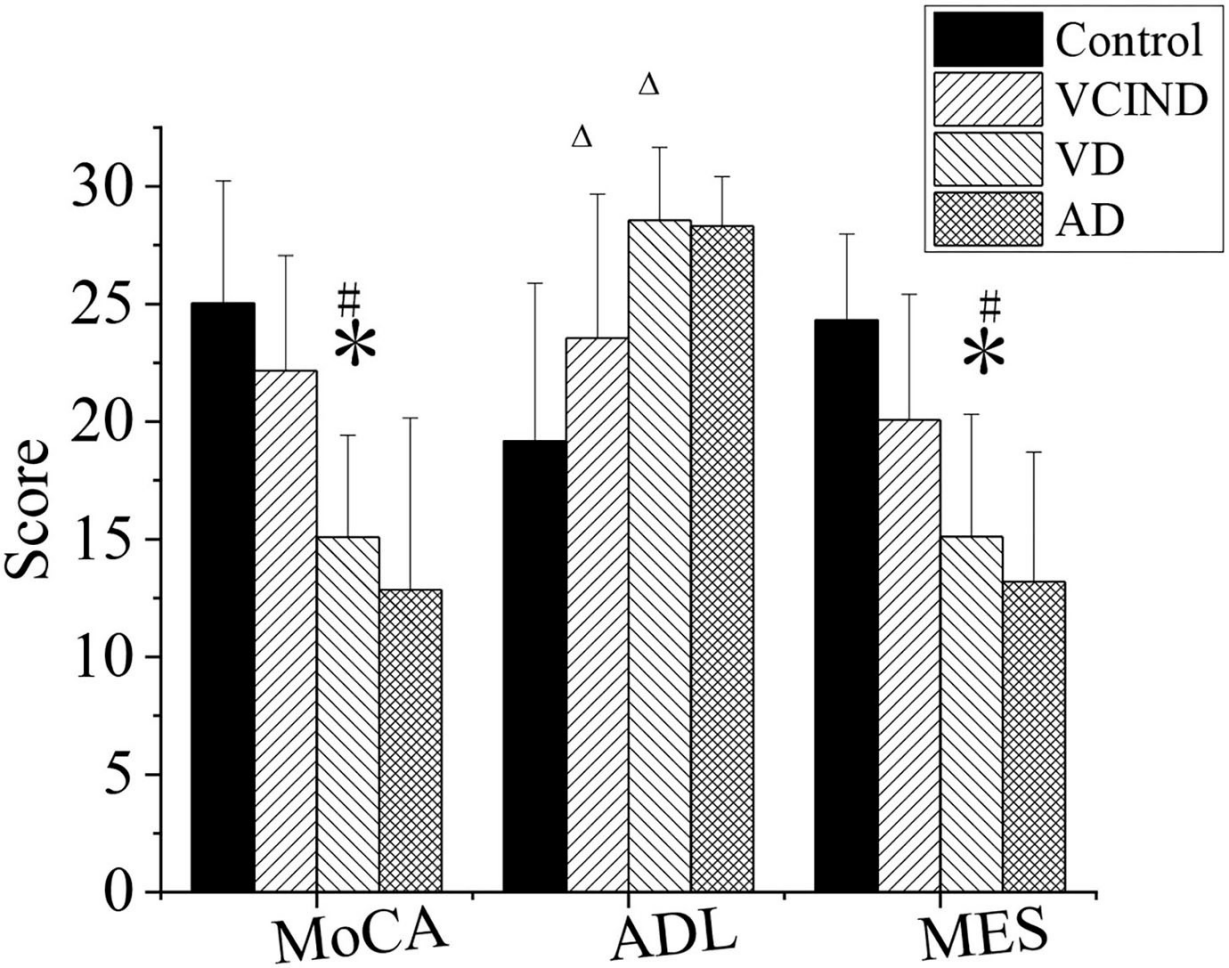
Comparison of cognitive functions of patients in the control, VCIND, VD, and AD groups. AD, Alzheimer's disease; VCIND, vascular cognitive impairment non-dementia; VD, vascular dementia. *p < 0.05 if compared with MMSE, MOCA, and MES in the control group, whereas ^#^*p* < 0.05 if compared with the VCIND group. ^Δ^*p* < 0.05 if compared with ADL in the control group.

MOCA, MES, and ADL were evaluated in the control, VCIND, VD, and AD groups, as shown in Figure 8.

First, in the MOCA and MES, the scores of patients in the VD group were 15.09 ± 4.33 and 15.11 ± 5.2, respectively, and were higher than those of the patients in the control group, which were MOCA (25.03 ± 5.2) and MES (24.31 ± 3.67) (^*^p < 0.05). The values in the VD group were also lower than those in the VCIND group, which were MOCA (22.17 ± 4.89) and MES (20.08 ± 5.33), with a statistical difference (^#^*p* < 0.05).

Second, in the ADL, the values in the VCIND group (23.55 ± 6.12) and the VD group (28.56 ± 3.1) were higher than that in the control group (19.17 ± 3.67), and it had a statistical difference (^Δ^*p* < 0.05). There was no significant difference compared between the VCIND group and the VD group (*p* > 0.05).

Third, there was no statistical difference for MOCA, MES, and ADL in the AD group from the control, VCIND, and VD groups (*p* > 0.05).

### Analysis of Distribution of Lacunar Cerebral Infarction and Number of Lesions of Patients in Four Groups

**Figure 9:**
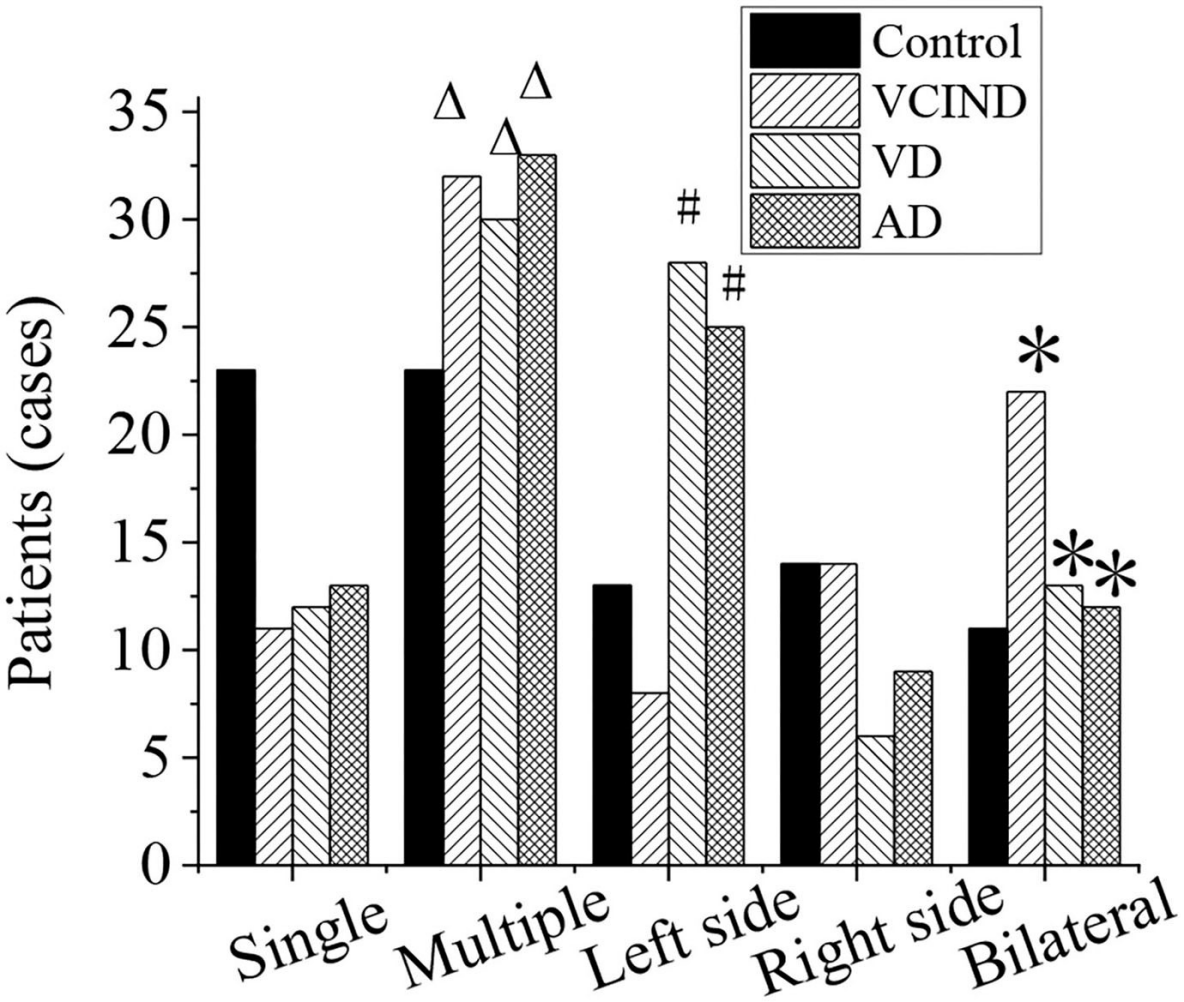
Analysis of distribution of lacunar cerebral infarction and number of lesions of patients in the control, VCIND, VD, and AD groups. AD, Alzheimer's disease; VCIND, vascular cognitive impairment non-dementia; VD, vascular dementia. *p < 0.05 if compared with distribution of lacunar cerebral infarction in the control group, whereas ^#^*p* < 0.05 if compared with the VCIND group. ^Δ^*p* < 0.05 if compared with number of lesions in the control group.

The distribution of lacunar infarction and the number of lesions of patients in the control group (46 cases), VCIND group (42 cases), VD group (42 cases), and AD group (46 cases) were analyzed, as shown in Figure 9. The ratios of the lacunar cerebral infarction distribution on both sides of the brain in the VCIND group (22 cases, 52.4%), VD group (13 cases, 31%), and AD group (12 cases, 26.1%) were significantly higher than that in the control group (11 cases, 23.9%), which had a statistical difference (^*^p < 0.05). The proportions of the lacunar cerebral infarction on the left side of the brain in the VD group (28 cases, 66.7%) and AD group (25 cases, 54.3%) were higher than that in the VCIND group (8 cases, 19%), which had a statistical difference (^#^*p* < 0.05).

The numbers of lacunar infarction lesions in the VCIND group (32 cases, 76.2%), VD group (30 cases, 71.4%), and AD group (33 cases, 71.7%) were significantly higher than that in the control group (23 cases, 50%), and it had a statistical difference (^Δ^*p* < 0.05).

### Analysis of White Matter Lesions and Incidence Rate of the Patients in Four Groups

**Figure 10:**
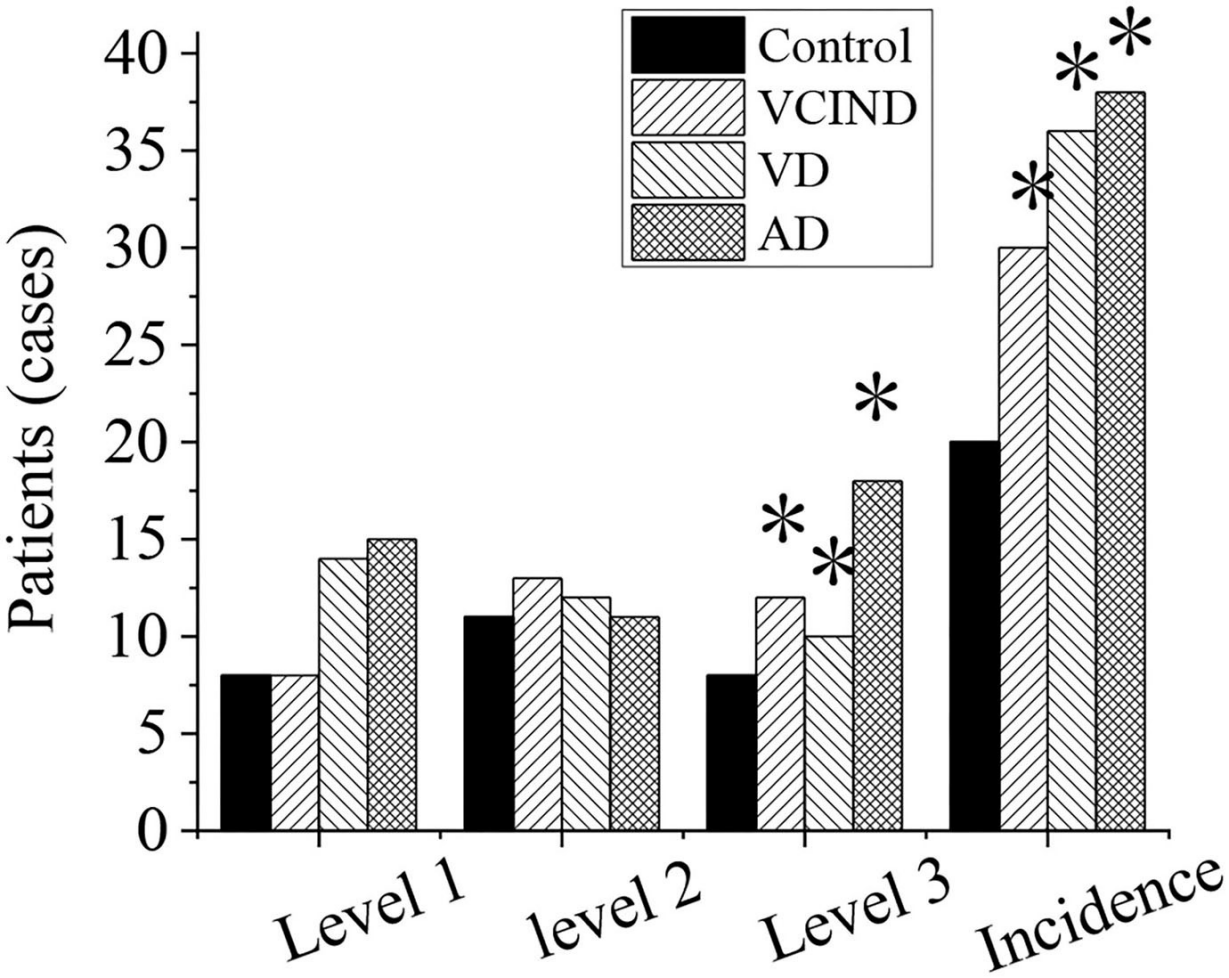
Analysis of white matter lesions and incidence rate of patients in the control, VCIND, VD, and AD groups. AD, Alzheimer's disease; VCIND, vascular cognitive impairment non-dementia; VD, vascular dementia. *p < 0.05 if compared with white matter lesions and incidence rate in the control group.

The white matter lesions and incidence rates of patients in the control group (46 cases), VCIND group (42 cases), VD group (42 cases), and AD group (46 cases) were compared and analyzed, as shown in Figure 10. Compared with the control group (8 cases, 17.4%), the proportions of 3-grade lesions in the VCIND group (12 cases, 28.6%), VD group (10 cases, 23.8%), and AD group (18 cases, 42.9%) were increased, which had a statistical difference (^*^p < 0.05). Compared with the control group (20 cases, 43.5%), the incidence rates of lesions in the VCIND group (30 cases, 71.4%), VD group (36 cases, 85.7%), and AD group (38 cases, 82.6%) were increased, which had statistical differences (^*^p < 0.05).

### Comparison of Hippocampal Volumes of Patients in Four Groups

**Figure 11:**
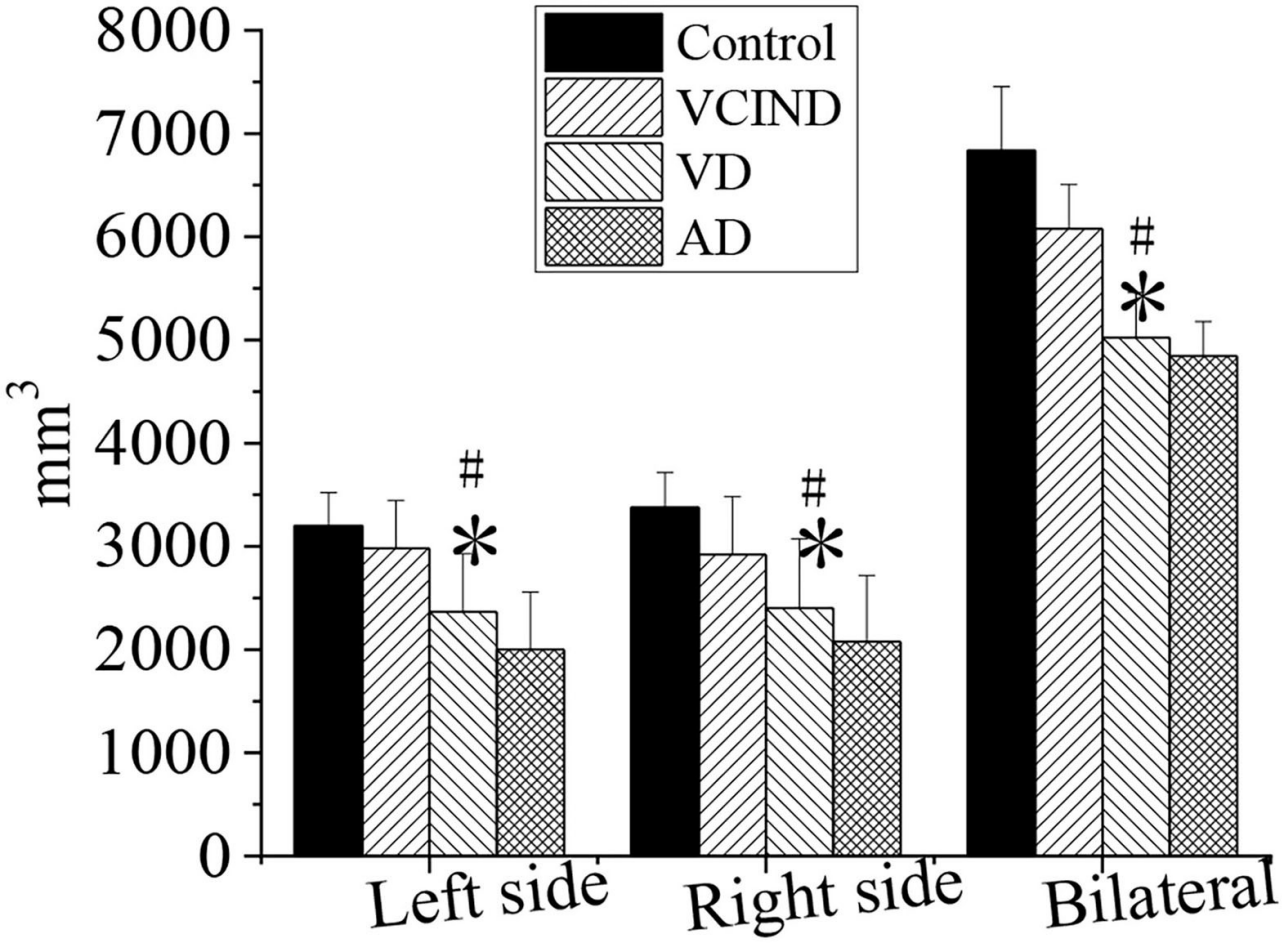
Comparison of hippocampal volumes of patients in the control, VCIND, VD, and AD groups. AD, Alzheimer's disease; VCIND, vascular cognitive impairment non-dementia; VD, vascular dementia. *p < 0.05 if compared with the left, right, and bilateral hippocampal volumes in the control group, whereas ^#^*p* < 0.05 if compared with the VCIND group.

The hippocampal volumes (left, right, and bilateral hippocampal volumes) of patients in the control, VCIND, VD, and AD groups were compared and analyzed, as shown in Figure 11. It compared the left (2,367 ± 563), right (2,401 ± 672), and bilateral hippocampal volume (5,023 ± 437) of patients in the VD group with the left (3,201 ± 321), right (3,378 ± 337), and bilateral hippocampal volume (6,834 ± 621) of patients in the control group. The results revealed that there was a statistical difference (^*^p < 0.05). By comparing the left (2,982 ± 463), right (2,921 ± 564), and bilateral hippocampus volume (6,079 ± 429) of the patients in the VCIND group, the left, right, and bilateral hippocampus volume of the patients in the VD group had a statistical difference (^#^*p* < 0.05).

### Relationship Between Hippocampal Volume and Age and Education Level

**Figure 12:**
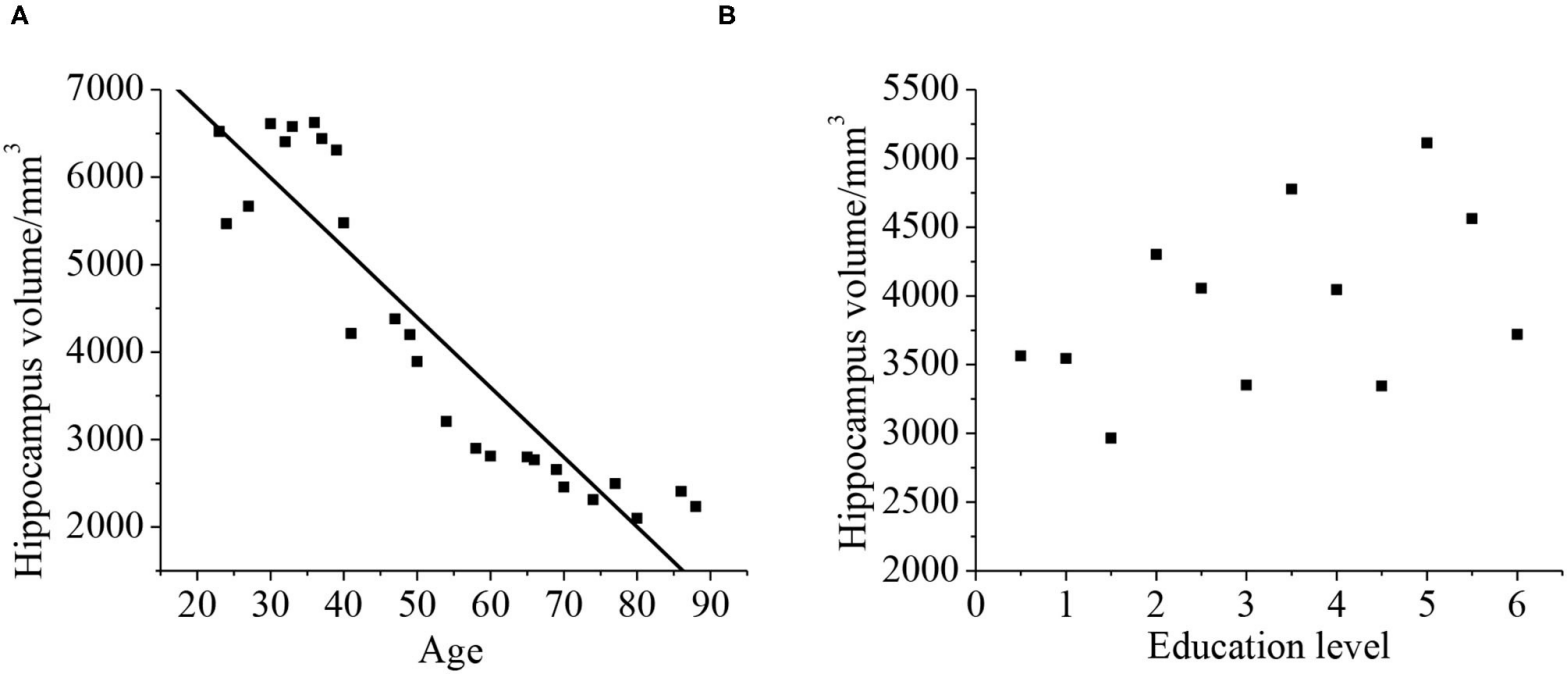
Correlation between the hippocampal volume, age **(A)**, and education level **(B)**. The correlation existed if *p* < 0.01.

As shown in Figure 12, Pearson's correlation analysis was taken for the relationship between the hippocampus volume and age, and it was found that the age and hippocampus volume were negatively correlated (*r* = −0.919, *p* < 0.01). With the increase of age, hippocampus volume tended to decrease. The hippocampal volume has no phase relationship with the education level (*p* > 0.01).

### Relationship Between Hippocampal Volume and the Neuropsychological Assessment ADL

**Figure 13:**
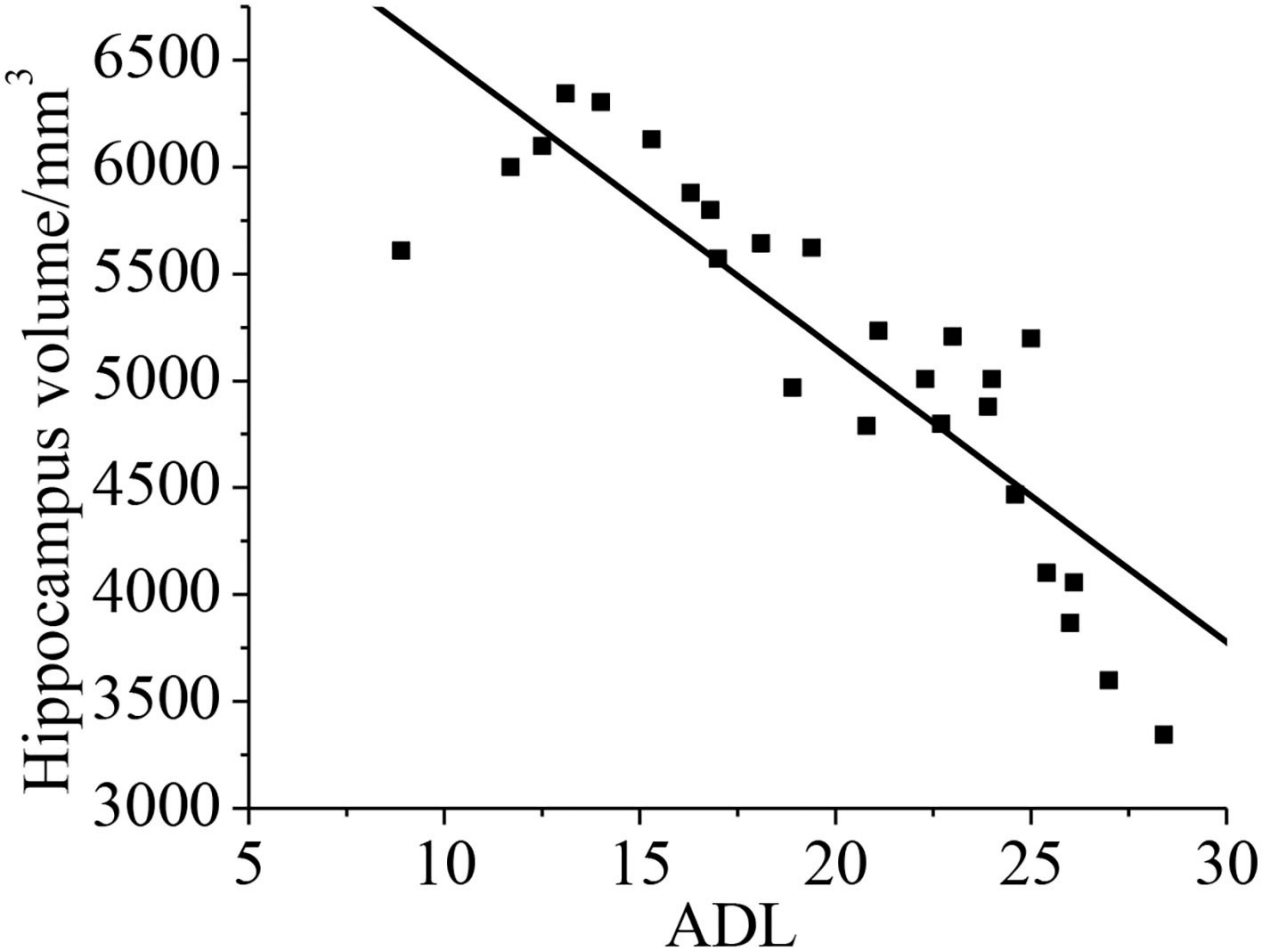
Correlation between the hippocampal volume ADL. The correlation existed if *p* < 0.01.

Pearson's correlation analysis was taken for the relationship between the hippocampal volume and neuropsychological assessment. It was found that the neuropsychological assessment ADL was negatively correlated with hippocampal volume (*r* = −0.872, *p* < 0.01). With less hippocampal volume of the patient, both the cognitive ability and daily living ability of the patient decrease, as shown in Figure 13.

## Discussion

Among fuzzy clustering, the most commonly used is FCM. However, FCM also has some shortcomings. The sum of the membership degrees of the same sample belonging to all classes is 1, which makes it sensitive to noise and outliers; and it is sensitive to the initial cluster center (18). In order to overcome these shortcomings, some scholars have proposed possible C-means clustering, weighted FCM clustering, and FCM based on the possibility of membership uncertainty. These clustering algorithms are based on the clustering of all special diagnoses in the dataset, and the weight of each special diagnosis is different (19, 20). In order to make the clustering results more accurate, the possibility of collaboration FCM is a method of clustering using the collaborative relationship between different features (12, 21). This method can be combined with other clustering algorithms to improve the clustering effect. The fuzzy partition matrix is obtained by using the synergy coefficient between the different features themselves (22). The detection by the MRI based on artificial intelligence technology will play an important role in the early diagnosis and treatment of diseases related to vascular cognitive impairment. Artificial intelligence can effectively complete the collection of relevant data information by simulating human thinking instead of an artificial collection. In this study, the relevant case data of patients in the Department of Neurology and Physical Examination who entered Changzhi People's Hospital from October 1, 2018, to February 1, 2020 were selected. Combined with the MRI data based on artificial intelligence technology, the risk factors of patients in the control, VCIND, VD, and AD groups were analyzed. It was found that the numbers of patients with associated hypertension in the VCIND group (52.4%), VD group (71.4%), and AD group (37%) are significantly higher than that in the control group (34.8%), which has a statistical difference (^*^p < 0.05). That in the VD group was significantly higher than that in the VCIND group, which had a statistical difference by comparison (^#^*p* < 0.05). There were no significant statistical differences among the other related risk factors (*p* > 0.05). It is consistent with the previous results, suggesting that it plays a key role in controlling hypertension in the prevention and control of cognitive impairment (23). If the blood pressure is under high pressure for a long time without any control, the elasticity of small cerebral blood vessels will be reduced accordingly, and the blood circulation will be poor. It will lead to hypoxia in the brain, lacunar cerebral infarction, and white matter lesions and develop into a VD. Some related studies show that diabetes, hyperlipidemia, and coronary heart disease are related to cognitive impairment (24), which is inconsistent with the results of this study. No correlation between gender, age, and education level to the cognitive impairment was found in this study.

In this study, a comprehensive evaluation of the cognitive ability is taken for all patients. It is found the scores of MOCA and MES in the VD group are 15.09 ± 4.33 and 15.11 ± 5.2, respectively. They are lower than that in the control group, and there is a statistical significance (*p* < 0.05). It indicates that compared with the control group, the cognitive level in the VD group decreases greatly, whereas the cognitive level in the VCIND decreases slightly. Such results are consistent with the study of Jadavji et al. (25). In the ADL, the scores in the VCIND group (23.55 ± 6.12) and the VD group (28.56 ± 3.1) are higher than that in the control group (19.17 ± 3.67), which has a statistical difference (*p* < 0.05). It means that compared with the control group, the daily living ability of patients in the VCIND group and the VD group are significantly decreased. The hippocampal volume is negatively correlated with ADL (*r* = −0.872, *p* < 0.01), indicating that the more serious the hippocampus atrophy, the worse the daily living ability of the patient. Such results are consistent with the results of Wierenga et al. (26). The hippocampal volume is negatively correlated with age (*r* = −0.919, *p* < 0.01), indicating that the hippocampus-related neurons may be lost as age increases, causing their volume to shrink.

The distribution of lacunar infarction and the number of lesions of the patients in the four groups were analyzed. It was found that the proportions of lacunar infarction in the brain (52.4, 31, and 26.1%) and the number of multiple lacunar infarction lesions (76.2, 71.4, and 71.7%) in VCIND, VD, and AD groups were significantly higher than those in the control group (23.9% and 50%). It has a statistical difference (*p* < 0.05). With the increase of the infarction, the incidence rate of the cognitive impairment also increases, and the degree of dementia increases. The bilateral and multiple lesions have more serious damage to the executive function of the patient. It may be due to the multiple arterial ischemias, and more neural network structures are also destroyed.

Compared with the control group, the proportions of 3-grade lesions (28.6, 23.8, and 42.9%) and the incidence rate of lesions (71.4, 85.7, and 82.6%) in the VCIND, VD, and AD groups increased, showing statistics differences. When the white matter of the brain suffers some lesion, the white matter fibers will be destroyed, and the cognitive function will be impaired. It is consistent with multiple studies that white matter lesions are related to cognitive impairment in patients with dementia and non-dementia.

## Conclusion

In this study, the lacunar cerebral infarction, white matter lesions, and hippocampal volume of the patient with the neurological cerebrovascular disease were studied, and the correlation with cognitive impairment of the patient was analyzed through MRI based on artificial intelligence technology combined with the neuropsychological assessment. However, the shortcoming of this study was that the patient samples involved were fewer, which may cause deviations in the results. Therefore, the results needed to be verified further by the multicenter experimental data with more samples. In short, the improved FCM algorithm had a higher segmentation effect and SA on MRI of neurovascular cerebrovascular diseases. The distribution, number, white matter lesions, and hippocampal volume of lacunar cerebral infarction were related to the cognitive impairment of patients with cerebrovascular diseases.

## Data Availability Statement

The original contributions presented in the study are included in the article/supplementary material, further inquiries can be directed to the corresponding author.

## Ethics Statement

The studies involving human participants were reviewed and approved by Changzhi Medical College. The patients/participants provided their written informed consent to participate in this study.

## Author Contributions

LZ wrote the manuscript. YL, LB, and QL collected and analyzed general data of patients. XZ and BZ were responsible for the follow-up index analysis. BZ helped with statistical analysis. All authors read and approved the final manuscript.

## Funding

This study was supported by the Scientific Research Project of Shanxi Provincial Health Commission in 2021 (No. 2021012) and the Key Special Project of Four Batches of Science and Technology Innovation Plan of Shanxi Provincial Health Commission (No. 2020XM38).

## Conflict of Interest

The authors declare that the research was conducted in the absence of any commercial or financial relationships that could be construed as a potential conflict of interest.

## Publisher's Note

All claims expressed in this article are solely those of the authors and do not necessarily represent those of their affiliated organizations, or those of the publisher, the editors and the reviewers. Any product that may be evaluated in this article, or claim that may be made by its manufacturer, is not guaranteed or endorsed by the publisher.
